# Metabolomics analysis of type 2 diabetes remission identifies 12 metabolites with predictive capacity: a CORDIOPREV clinical trial study

**DOI:** 10.1186/s12916-022-02566-z

**Published:** 2022-10-27

**Authors:** Marina Mora-Ortiz, Juan F. Alcala-Diaz, Oriol Alberto Rangel-Zuñiga, Antonio Pablo Arenas-de Larriva, Fernando Abollo-Jimenez, Diego Luque-Cordoba, Feliciano Priego-Capote, Maria M. Malagon, Javier Delgado-Lista, Jose M. Ordovas, Pablo Perez-Martinez, Antonio Camargo, Jose Lopez-Miranda

**Affiliations:** 1 Lipids and Atherosclerosis Unit, Internal Medicine Unit, Reina Sofia University Hospital, 14004 Cordoba, Spain; 2 Department of Medical and Surgical Science, University of Cordoba, 14004 Córdoba, Spain; 3 Maimonides Biomedical Research Institute of Cordoba (IMIBIC), Av. Menendez Pidal, s/n, 14004 Cordoba, Spain; 4 CIBER Fisiopatologia de la Obesidad y Nutricion (CIBEROBN), Instituto de Salud Carlos III, 28029 Madrid, Spain; 5 Department of Analytical Chemistry and Nanochemistry University Institute, Universidad de Cordoba, Cordoba, Spain; 6 CIBER de Fragilidad Y Envejecimiento Saludable (CIBERFES), Instituto de Salud Carlos III, Madrid, Spain; 7 Department of Cell Biology, Physiology and Immunology, University of Cordoba, 14004 Cordoba, Spain; 8 Nutrition and Genomics Laboratory, J.M.-US Department of Agriculture Human Nutrition Research Center On Aging at, Tufts University, Boston, MA 02111 USA; 9 IMDEA Alimentacion, Madrid, Spain; 10 CNIC, 28049 Madrid, Spain

**Keywords:** Diabetes, Insulin resistance, Prospective human study, Metabolomics

## Abstract

**Background:**

Type 2 diabetes mellitus (T2DM) is one of the most widely spread diseases, affecting around 90% of the patients with diabetes. Metabolomics has proven useful in diabetes research discovering new biomarkers to assist in therapeutical studies and elucidating pathways of interest. However, this technique has not yet been applied to a cohort of patients that have remitted from T2DM.

**Methods:**

All patients with a newly diagnosed T2DM at baseline (*n* = 190) were included. An untargeted metabolomics approach was employed to identify metabolic differences between individuals who remitted (RE), and those who did not (non-RE) from T2DM, during a 5-year study of dietary intervention. The biostatistical pipeline consisted of an orthogonal projection on the latent structure discriminant analysis (O-PLS DA), a generalized linear model (GLM), a receiver operating characteristic (ROC), a DeLong test, a Cox regression, and pathway analyses.

**Results:**

The model identified a significant increase in 12 metabolites in the non-RE group compared to the RE group. Cox proportional hazard models, calculated using these 12 metabolites, showed that patients in the high-score tercile had significantly (*p*-value < 0.001) higher remission probabilities (Hazard Ratio, HR, _high versus low_ = 2.70) than those in the lowest tercile. The predictive power of these metabolites was further studied using GLMs and ROCs. The area under the curve (AUC) of the clinical variables alone is 0.61, but this increases up to 0.72 if the 12 metabolites are considered. A DeLong test shows that this difference is statistically significant (*p*-value = 0.01).

**Conclusions:**

Our study identified 12 endogenous metabolites with the potential to predict T2DM remission following a dietary intervention. These metabolites, combined with clinical variables, can be used to provide, in clinical practice, a more precise therapy.

**Trial registration:**

ClinicalTrials.gov, NCT00924937.

**Supplementary Information:**

The online version contains supplementary material available at 10.1186/s12916-022-02566-z.

## Background

T2DM is a metabolic disorder widely identified by a generalized hyperglycaemia and insulin resistance [1]. Nowadays, approximately 422 million adults are diagnosed with diabetes [2]; T2DM is the most prevalent form of the disease, affecting 90% of the patients (circa 380 million worldwide). Common co-morbidities associated with T2DM include cardiovascular diseases, blindness, nerve damage, and kidney failure [3–5]. The co-occurrence of coronary heart disease (CHD) along with T2DM markedly increases the risk of macrovascular complications and mortality. Indeed, macrovascular events represent circa 80% of all deaths in these patients [1]. This current scenario urges us to find new approaches to diagnose and treat these patients. Metabolic profiling, or metabolomics, allows for the characterization of hundreds of compounds (i.e. metabolites) facilitating functional information of the metabolism at the time when the sample is taken [6–8] and has proven useful to understand the metabolism in the context of (i) diagnostic and prognostic biomarker discovery, (ii) therapy research, and (iii) pathways determination within T2DM [8].

Recent evidence has shown that T2DM is reversible when tackled in an early phase following two different strategies: calorie restriction and bariatric surgery. The former is linked to weight loss, gut permeability, and reduction in inflammatory and endotoxemia biomarkers [9]. The latter leads to normalizing plasma glucose levels and a significant weight loss [10–12].

Here, we analyse the metabolomics modulations differences between individuals where T2DM has remitted (RE) and those who did not recover and consequently remained as diabetics (non-RE) after 5 years of dietary intervention. The dietary intervention consisted of two different types of diets: low-fat diet (LF) and Mediterranean diet (MED).

## Methods

### Aim and objectives

The aim is to characterize the metabolic profile of non-RE and RE patients in serum samples at the baseline to identify biomarkers of interest to assist in the diagnosis, monitoring, and treatment of the disease. These biomarkers can be particularly useful to predict who will recover from T2DM following a dietary intervention.

The specific objectives of our study are to analyse the metabolomics differences before the dietary intervention and identify biomarkers of interest to assist in the prediction of T2DM recovery.

### Study design and participants

This study was developed within the framework of the CORDIOPREV (CORonary Diet Intervention with Olive oil and cardiovascular PREVention) study, registered at Clinicaltrials.gov (number NCT00924937). This study is an ongoing controlled, single-blind, and randomized trial, with 1002 CHD patients. The trial protocol and subsequent revisions were approved by the Reina Sofia University Hospital Ethics Committee, following the Helsinki Declaration and good clinical practices. All patients signed a written informed consent to participate in the study.

Patients’ recruitment took place between November 2009 and February 2012, mostly at Reina Sofia University Hospital, Córdoba, Andalusia, Spain, with contributions from other hospitals in Córdoba and Jaen areas, in Andalusia, Spain. Complete details of the study methods, rationale, inclusion criteria, cardiovascular risk factors, and baseline characteristics are found elsewhere [13]. In brief, eligible participants, in the age range of 20 to 75 years, had established CHD with no clinical events in the previous 6 months. They all had at least a 5-year life expectancy and no other concurrent major diseases and were willing to participate in a long-term monitoring study [13].

**Fig. 1:**
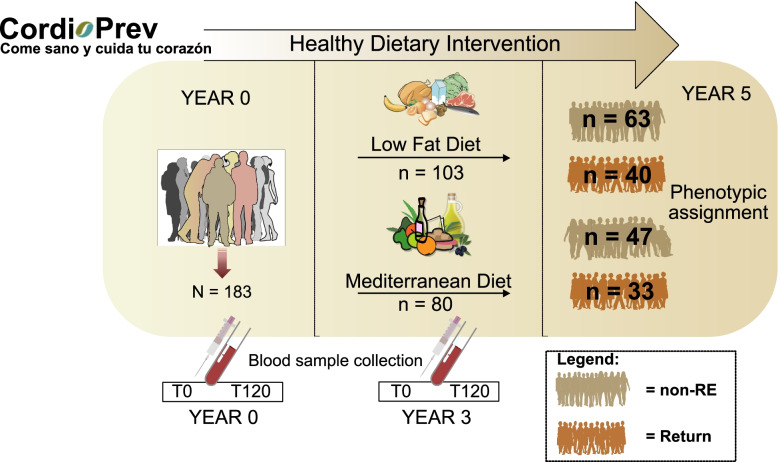
CORDIOPREV study design

In this work, 183 patients, from the CORDIOPREV study (https://www.cordioprev.es/index.php/es/) diagnosed with diabetes, underwent a dietary interventional study where participants were offered either an LF or MED diet for 5 years (Fig. 1). In our study, blood samples were taken during fasting (time 0) and 120 min after a glucose boost.

### Oral glucose tolerance test

The patients underwent an OGTT at the baseline and, once a year, every year during the dietary intervention. Before the test, patients had fasted (from food/drugs) for 12 h and were asked to refrain from smoking and alcohol intake during the preceding 7 days. They were also asked to avoid strenuous physical activity, a day before the test. At 8:00 A.M., patients were admitted to the laboratory to perform the oral glucose tolerance test (OGTT) (75 g flavoured glucose load, Trutol 75; Custom Laboratories, Baltimore, MD, USA). Blood samples were taken at times corresponding to 0, 30, 60, 90, and 120 min to determine the glucose and insulin concentrations [14]. The insulin sensitivity index (ISI) was calculated from the OGTT using the following formula: ISI = 10.000 ÷ √([fasting plasma glucose X fasting plasma insulin] × [mean glucose in OGTT × mean insulin in OGTT]) [14]. HOMA-IR was calculated as described by Song et al. [15]. Beta-cell function was calculated using the disposition index (DI) as follows: DI = ISI × [AUC30 min insulin/AUC30 min glucose], where AUC30 min is the area under the curve between baseline and that at 30 min of the OGTT for insulin (pmol/l) and glucose (mmol/l) measurements, calculated by the trapezoidal method [16]. The indices used to determine tissue-specific insulin resistance (IR) were the hepatic insulin resistance index (HIRI, fasting plasma insulin × fasting plasma glucose) and the muscle insulin sensitivity index (MISI, (dG/dt)/mean of plasma insulin) [17]. Insulinogenic Index (IGI) was calculated by measuring plasma insulin at 30 min − fasting plasma insulin (mU/L)/(plasma glucose at 30 min − fasting plasma glucose(mg/dL) [18].

The adipose tissue (AT) insulin resistance index (Adipo-IR) was determined according to the formula: Adipo-IR = fasting plasma NEFA (mM) × fasting plasma insulin (pmol/L), which has been found as a suitable and useful method in clinical practice to estimate AT insulin sensitivity [19].

### Randomization and masking

The process of randomization has been reported elsewhere [13]. Briefly, this is based on the following variables: sex (male, female), age (under and over 60 years old), and previous myocardial infarction (yes, no). Eight different groups were created to represent all the possible combinations of the above factors. Therefore, eight different blocks were created to assign the diets (bloc randomization). Dietitians were the only members of the intervention team to be aware of the dietary group of each participant.

### Dietary assessment

The participants were randomized to consume two diets: the Med diet or an LF diet [13]. The LF diet consists of < 30% total fat (< 10% saturated fat, 12–14% MUFA fat, and 6–8% PUFA fat), 15% protein, and a minimum of 55% carbohydrates. The Med diet consists of a minimum of 35% of calories as fat (22% MUFA fat, 6% PUFA fat, and < 10% saturated fat), 15% proteins, and a maximum of 50% carbohydrates [20]. Neither energy restriction, nor physical activity was specifically encouraged. In both diets, the cholesterol content was adjusted to < 300 mg/d.

The Mediterranean and low-fat diets were designed to provide a wide variety of foods, including vegetables, fruit, cereals, potatoes, legumes, dairy products, meat, and fish. The participants in both intervention groups received the same intensive dietary counselling. The nutritionists administered personalized individual advice every 6 months. In addition, quarterly group education sessions were held with up to 20 participants per session; separate group sessions were performed every 3 months, and dietary counselling by phone was done every 2 months [20]. At the beginning of the study, and every 6 months afterwards, each patient had a face-to-face interview with a nutritionist to complete a 137-item semi-quantitative food frequency questionnaire (validated in Spain [21]). The dietary evaluation was calculated by the 14-item Med Diet Adherence Screener, which was used for measuring adherence to the Med diet [22]. Moreover, a 9-item dietary adherence screener was used to measure adherence to the LF diet guidelines. A more detailed report on dietary adherence has been published recently by our research group [20].

### Diabetes remission criteria

Remission required the following: (i) the absence of glucose-lowering treatment and was defined by levels of HbA1c < 6·5%, (ii) a fasting plasma glucose < 126 mg/dl, and (iii) a 2-h plasma glucose in the 75 g OGTT < 200 mg/dl maintained for at least 2 years. This agrees with the American Diabetes Association (ADA) diagnosis criteria [23].

### Sample preparation

Plasma samples (100 μL) were immersed in bath ice and treated with 300 μL of 3:1 (v/v) methanol–acetonitrile (MeOH–ACN). The treated samples were vortexed for 2 min and subsequently cooled at − 20 °C for 3 min. Centrifugation was carried out for 15 min at 4 °C and 13,800 × g in a thermostatic centrifuge Thermo Sorvall Legend Micro 21 R from Thermo (Thermo Fisher Scientific, Bremen, Germany), and the supernatant phase was isolated. This phase was dried by evaporation and reconstituted with 60 μL of 3:1 (v/v) MeOH–ACN. All samples were processed in a 1200 Series LC system (Agilent Technologies, Waldbronn, Germany) coupled to an Agilent 6530 high-resolution QTOF mass spectrometer equipped with a dual electrospray ionization source.

### LC–QTOF MS/MS analysis

A Poroshell 120 EC-C18 column (50 mm × 2.1 mm i.d., 2.7 μm particle size, from Agilent), kept at 25 °C, was used to carry out the chromatographic division. The mobile phases consisted of (A) 0.1% formic acid in deionized water and (B) 0.1% formic acid in acetonitrile. The protocol used for the elution consisted of 0–2 min, 5% B; 2–11 min and the percentage of mobile phase B was modified from 0 to 100%. The final percentage was held for 6 min. Five minutes post-run was included to equilibrate the column. The flow rate was maintained at 0.4 mL/min. The injected sample volume was 5.0 μL and the injector needle was washed 10 times with 70% methanol between injections. Therefore, the needle seat was flushed for 15 s at a flow rate of 4 mL/min, with 70% methanol, to avoid cross-contamination between samples. The autosampler was kept at 4 °C to increase sample stability. The settings of the electrospray ionization source, which was operated in negative and positive ionization modes, were as follows: capillary voltage ± 3.5 kV, Q1 voltage 130 V, N2 pressure in the nebulizer 35 psi; N2 flow rate and temperature as drying gas 10 L min–1 and 325 °C, respectively. MS/MS data were acquired in both polarities, using the centroid mode at a rate of 2.5 spectra s–1 in extended dynamic range mode (2 GHz). Accurate mass spectra in the MS scan were acquired in the m/z range 40–1100 and the MS/MS mode in the m/z range 30–1100. The instrument gave a typical resolution of 18,000 full width at half maximum (FWHM) at m/z 118.0862 and 35,000 FWHM at m/z 922.0098. The instrument was calibrated and tuned as recommended by the manufacturer. To assure the desired mass resolution, continuous internal calibration was performed during analyses by using the signals at m/z 121.0509 (protonated purine) and m/z 922.0098 [protonated hexakis(1H,1H,3H-tetrafluoropropoxy) phosphazine or HP-921] in the positive ion mode, while in the negative ion mode, ions with m/z 119.0362 (proton abstracted purine) and m/z 966.0007 (formate adduct of HP-921) were used. The collision energy was set at 20 V for the whole run. The analytical samples were injected in auto MS/MS acquisition mode to obtain fragmentation information from a maximum of two precursors selected per cycle with an exclusion window of 0.1 min after 2 consecutive selections of the same precursor.

### Data processing

The MassHunter Workstation software (version B7.00 Qualitative Analysis, Agilent Technologies, Santa Clara, CA, USA) was used to process all the data obtained by LC–QTOF in data-dependent acquisition MS/MS mode. Treatment of raw data files started with the extraction of potential molecular features (MFs) with the suited algorithm included in the software. For this aim, the extraction algorithm considered all ions exceeding 500 counts for both polarities with a maximum charge state of 2 for the obtained chromatograms. The count cut-off value was established considering the chromatographic background noise. Additionally, only MFs defined by two or more ions were considered, with a tolerance for the isotopic distribution of 0.0025 m/z for peak spacing tolerance, plus 7.0 ppm in mass accuracy. Only the following potential ions and adducts were considered in positive (H + , Na + , K + , NH4 +) and negative ionization (H − , HCOO + , Cl +) modes. Furthermore, a potential neutral loss by dehydration was also included to identify features corresponding to the same potential metabolite.

Identification of metabolites was supported on MS and MS/MS information by using METLIN MS and MS/MS databases (http://metlin.scripps.edu), the Human Metabolome Database (HMDB, 3.6 version), and the LIPID MAPS website ((http://www.lipidmaps.org); in all cases, the MFs obtained in the previous step were used. A database with all identified metabolites was used to perform a targeted compound extraction analysis using a tolerance window of 0.8 min and 5 ppm mass accuracy. This step was performed with Profinder Analysis (version B8.00, Agilent Technologies, Santa Clara, CA, USA). A table with the peak area of all identified compounds in the different samples injected was obtained as a result.

### Statistical analysis

Metabolites showing in at least 80% of the samples were selected for further analysis. To allow predictive modelling, imputation was carried out when needed, substituting missing values by half the smallest value of the appropriate metabolite.

LC–MS data (polar and apolar) was imported into Matlab (R2015a, Mathworks UK) and analysed using the statistic toolbox and algorithms from Korrigan Toolbox version 0.1 (Korrigan Sciences Ltd, UK). In Matlab, matrices were log 10 normalized. The biostatistical pipeline for the multivariate statistical analysis considered a preliminary unsupervised principal component analysis (PCA), followed by a supervised pairwise O-PLS DA [24, 25], which identifies the specific modulations driven by the appropriate predictor (i.e. individuals who returned from T2DM versus those who did not).

To assess the predictive power of the O-PLS DA models, *R*^*2*^ (the explained variance) was calculated. This parameter evaluates the model maximizing variance given by the endogenous variables. The *Q*^*2*^, or goodness of prediction, assesses the predictive relevance of the model and is based on a matrix partition technique that ignores part of the data (in our case a seventh part each time), estimates the model parameters, and predicts the omitted parts using the estimates obtained previously. *Q*^*2*^ greater than 0 means the model has predictive value. In addition, the overfitting of the model (the difference between *R*^*2*^Y and *Q*^*2*^Y) was also considered, and only models with less than 50% overfitting were further considered. Model parameters and associated metabolites were reported and used for a Cox proportional hazard model, GLM, and ROC calculations in R (version 4.0.5 (2021–03-31, https://www.r-project.org) using the packages ‘caret’ and ‘pROC’. Unadjusted Cox proportional-hazard models calculated the hazard ratio (HR) of every metabolite previously identified in the O-PLS DA model within a 95% confidence interval (CI). This unadjusted Cox allowed the identification of the betas for every metabolite. This information was used to calculate the patient’s likelihood of recovering from diabetes by running a Cox analysis adjusted for sex, age, body mass index (BMI), HDL, triglycerides, and intensity of statin therapy based on the tertiles resulting from the multiplication of the betas previously obtained by the abundance of each metabolite for every patient. Finally, generalized linear models were calculated for (i) all the clinical variables (i.e. sex, age, BMI, HDL, triglycerides, and intensity statin therapy), (ii) all the metabolites, (iii) the glycated haemoglobin, (iv) the clinical variables and the 12 metabolites, and (v) the clinical variables and the glycated haemoglobin. ROC analyses were carried out for these three models, and AUC, sensitivity, specificity, accuracy, and threshold were estimated for the models. Finally, DeLong analysis was used to compare whether the AUCs of these models were or not significantly different between them.

## Results

### Baseline characteristic

BMI, waist circumference, body weight, glucose, glycated haemoglobin (HbA1c), insulin, HIRI, and homeostatic model assessment of insulin resistance (HOMA-IR) were statistically significantly higher at baseline in the non-RE group than in the RE group. Conversely, ISI and DI values were statistically significantly lower at baseline in the RE group than in the non-RE group (*p* < 0.05) (Additional file 1: Table S1).

### O-PLS DA results from the comparison between individuals who remitted from T2DM and those who did not

**Fig. 2:**
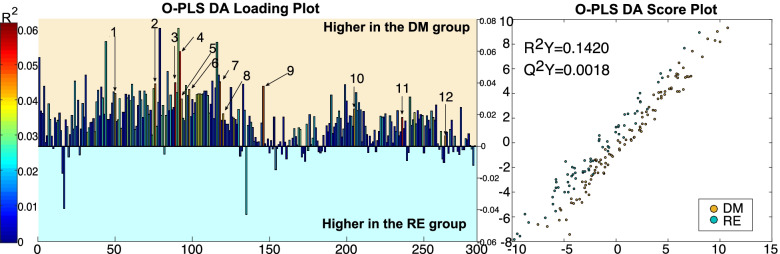
O-PLS DA analysis loading and score plot calculated using all spectra as a matrix of independent variables and diabetic remission as the predictor

The O-PLS DA was examined based on *R*^*2*^*Y*, *Q*^*2*^*Y*, and overfit parameters obtained in every pairwise comparison (see material and methods). The O-PLS DA analysis identified differences between RE and DM individuals during fasting (*R*^*2*^*Y* = 0.1420, *Q*^*2*^*Y* = 0.0018), but not after a glucose overdose (Fig. 2). DM individuals had higher levels of sphingosine (d18:2), docosenamide, oxo-tricosanoic acid, tetracosahexaenoic acid, ketodeoxycholic acid, stearoylcarnitine, diglyceride (33:4), creatine, tridecanoic acid, monoacylglycerol (22:6), dihydroxycholesterol, and biliverdin. These metabolites, as well as a ranking with the degree of statistically significant association of each metabolite, are presented in further detail in Additional file 2: Fig. S1.

### Cox proportional hazard models

**Fig. 3:**
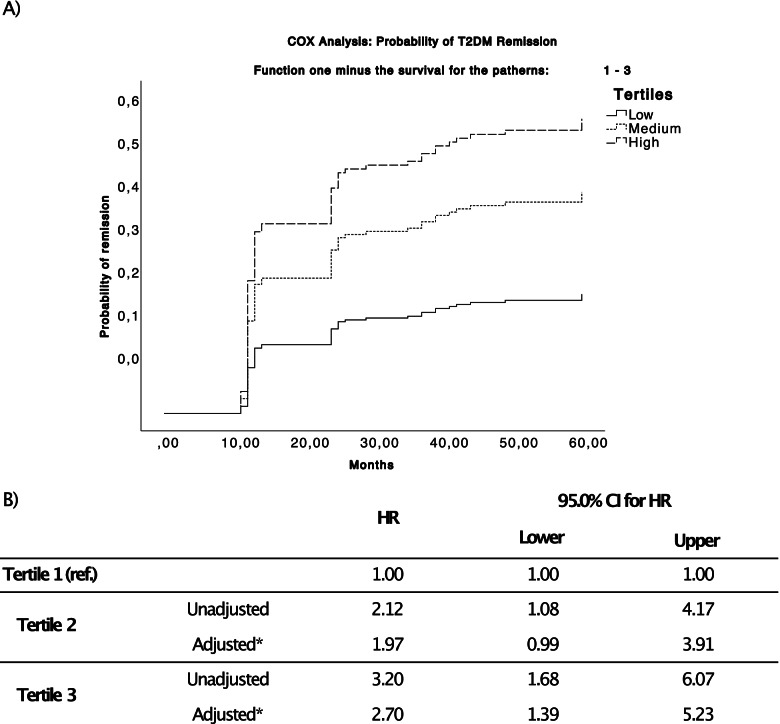
Adjusted Cox for the analysis of the patient risk scored grouped in tertiles. **a** Survival probability chart overtime (expressed in months). **b** Hazard ratio of the risk score for the three tertiles and the covariables

Adjusted and unadjusted Cox proportional hazard models were calculated for every metabolite previously identified in the O-PLS DA model. These metabolites were grouped in terciles for the Cox analysis. The results are shown in Table 1. The unadjusted beta values were used to calculate the patient risk score (beta × metabolite abundance) for all the patients and metabolites. This patient risk score grouped by terciles was used to carry out a new Cox model adjusted for sex, age, BMI, HDL, TG, and intensity statin therapy (see Fig. 3). Results from the Cox analysis of the patient’s risk score showed that individuals in the high tercile (luckily to recover from T2DM) had an HR of 2.70 compared to individuals in the low tercile; this difference was statistically significant (*p*-value = 0.003). Moreover, we also grouped the patients by ascending terciles of glycated haemoglobin, to carry out a Cox model adjusted for sex, age, BMI, HDL, TG, and intensity statin therapy (Additional file 2: Fig. S2). Results from the Cox analysis of the glycated haemoglobin showed that individuals in the low and medium tercile had an HR of 3.80 and 4.31 respectively, compared to individuals in the High tercile (both *p*-value = 0.001).

**Table 1:** Cox proportional hazard models were calculated for every metabolite. The hazard ratios (HR) between groups were calculated with Tertile 3 as a reference and adjusted by age, gender, diet, body mass index, HDL-c, and triglycerides

					**95.0% CI for HR**	
**Metabolite**	**Tertile**	**Model type**	**Sig**	**HR**	**Lower**	**Upper**
**Sphingosine (d18:2)**	Tertile 3 (ref.)					
	Tertile 2	Unadjusted	.283	1.414	.751	2.664
		Adjusted	.324	1.389	.723	2.667
	Tertile 1	Unadjusted	.013	2.111	1.171	3.806
		Adjusted	.018	2.059	1.129	3.753
**Docosenamide**	Tertile 3 (ref.)					
	Tertile 2	Unadjusted	.704	1.122	.621	2.027
		Adjusted	.670	1.141	.622	2.094
	Tertile 1	Unadjusted	.320	1.336	.755	2.364
		Adjusted	.404	1.278	.718	2.275
**Oxo-tricosanoic acid**	Tertile 3 (ref.)					
	Tertile 2	Unadjusted	.522	1.222	.661	2.257
		Adjusted	.458	1.265	.680	2.352
	Tertile 1	Unadjusted	.046	1.794	1.009	3.189
		Adjusted	.044	1.832	1.017	3.300
**Tetracosahexaenoic acid**	Tertile 3 (ref.)					
	Tertile 2	Unadjusted	.008	2.440	1.258	4.732
		Adjusted	.004	2.758	1.384	5.496
	Tertile 1	Unadjusted	.002	2.829	1.479	5.413
		Adjusted	.001	3.155	1.571	6.336
**Ketodeoxycholic acid**	Tertile 3 (ref.)					
	Tertile 2	Unadjusted	.612	.850	.453	1.595
		Adjusted	.677	.871	.456	1.665
	Tertile 1	Unadjusted	.065	1.682	.969	2.920
		Adjusted	.065	1.717	.967	3.046
**Stearoylcarnitine**	Tertile 3 (ref.)					
	Tertile 2	Unadjusted	.039	1.921	1.034	3.568
		Adjusted	.050	1.890	1.001	3.570
	Tertile 1	Unadjusted	.039	1.911	1.033	3.533
		Adjusted	.063	1.820	.967	3.426
**Diglyceride (33:4)**	Tertile 3 (ref.)					
	Tertile 2	Unadjusted	.569	1.207	.632	2.304
		Adjusted	.464	1.279	.661	2.474
	Tertile 1	Unadjusted	.004	2.380	1.328	4.266
		Adjusted	.004	2.423	1.321	4.444
**Creatine**	Tertile 3 (ref.)					
	Tertile 2	Unadjusted	.115	1.635	.887	3.014
		Adjusted	.123	1.632	.876	3.042
	Tertile 1	Unadjusted	.061	1.778	.973	3.249
		Adjusted	.093	1.703	.916	3.166
**Tridecanoic acid**	Tertile 3 (ref.)					
	Tertile 2	Unadjusted	.327	1.372	.729	2.585
		Adjusted	.394	1.323	.695	2.518
	Tertile 1	Unadjusted	.013	2.101	1.166	3.786
		Adjusted	.033	1.946	1.053	3.594
**Monoacylglycerol (22:6)**	Tertile 3 (ref.)					
	Tertile 2	Unadjusted	.315	1.358	.748	2.466
		Adjusted	.406	1.311	.692	2.485
	Tertile 1	Unadjusted	.131	1.571	.873	2.827
		Adjusted	.109	1.634	.896	2.979
**Dihydroxycholesterol**	Tertile 3 (ref.)					
	Tertile 2	Unadjusted	.388	.744	.381	1.454
		Adjusted	.373	.736	.375	1.445
	Tertile 1	Unadjusted	.008	2.099	1.214	3.631
		Adjusted	.019	1.987	1.119	3.529
**Biliverdin**	Tertile 3 (ref.)					
	Tertile 2	Unadjusted	.842	1.064	.577	1.964
		Adjusted	.961	.984	.517	1.872
	Tertile 1	Unadjusted	.116	1.574	.894	2.772
		Adjusted	.168	1.497	.843	2.657

### Generalized linear models and receiving operating characteristics

**Fig. 4:**
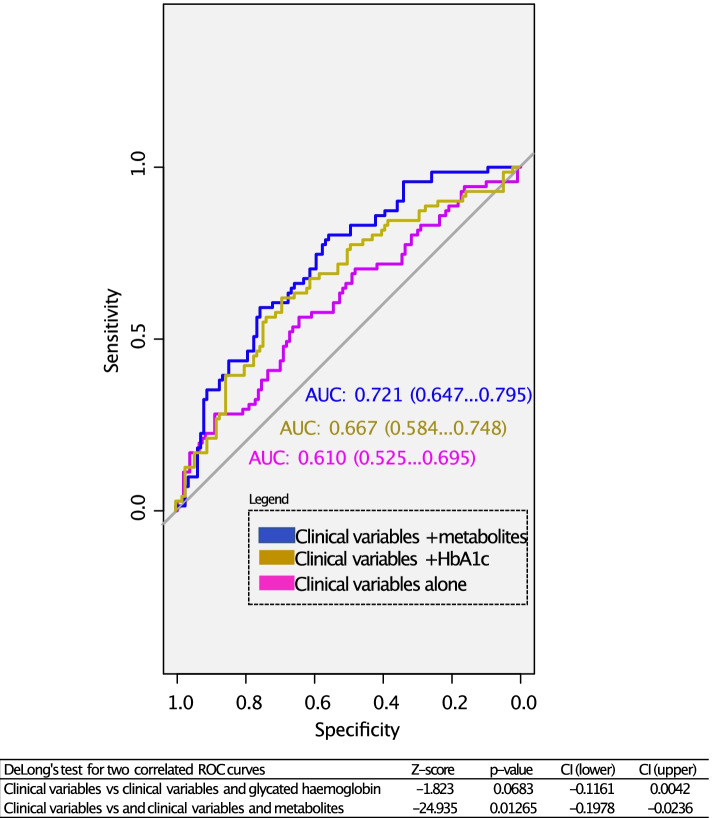
ROCs were obtained for the clinical variables and in combination with the metabolites or glycated haemoglobin (HbA1c)

GLMs and ROCs were run for (i) clinical variables alone, (ii) the 12 metabolites of interest, (iii) glycated haemoglobin, (iv) the clinical variables and the 12 metabolites, and (v) the clinical variables and the glycated haemoglobin. AUC, sensitivity, specificity, accuracy, and threshold were calculated in all cases. The results are shown in Tables 2 and 3 and Fig. 4. The AUC for the clinical variables was 0.610, for the metabolites was 0.701, for the glycated haemoglobin 0.618, for the combination of clinical variables and the 12 metabolites 0.721, and for the combination of the clinical variables and the glycated haemoglobin 0.667. A DeLong test was carried out comparing the AUC from the GLM of the clinical variables alone with the GLM with the clinical variables and the 12 metabolites or the GLM with the clinical variables and the glycated haemoglobin (Fig. 4). There were not significant differences between the model with the clinical variables alone and the addition of the glycated haemoglobin. However, the *p*-value (0.01265) resulting from the DeLong test comparing the model with the clinical variables alone and the clinical variables with the 12 metabolites indicated that these two models were significantly different and showed that the addition of the metabolites significantly improved the prediction capacity of the model.

**Table 2:** Results were obtained for the generalized linear models used with the (i) clinical variables, (ii) metabolites, and (iii) clinical variables and metabolites. * means statistically significant

		**Estimate**	**Std. error**	***z* value**	**Pr( >|*z*|)**
Clinical variables	Intercept	− 0.43249	0.18301	− 2.363	0.0181*
	Sex	− 0.08264	0.16283	− 0.508	0.6118
	Age baseline	0.09660	0.16102	0.600	0.5486
	HDL Imputed	0.10651	0.16663	0.639	0.5227
	TG Imputed	− 0.27225	0.17275	− 1.576	0.1150
	BMI baseline	0.44999	131.228	0.343	0.7317
	Intensity statin therapy	− 0.11911	0.15505	− 0.768	0.4424
Metabolites	Intercept	− 0.459189	0.704513	− 0.652	0.5145
	Sphingosine (d18:2)	− 0.107924	0.260230	− 0.415	0.6783
	Docosenamide	0.004636	0.004572	1.014	0.3106
	Oxo-tricosanoic acid	− 0.002283	0.003195	− 0.714	0.4750
	Tetracosahexaenoic acid	− 0.003083	0.003112	− 0.991	0.3219
	Ketodeoxycholic acid	− 0.293914	0.290994	− 1.010	0.3125
	Stearoylcarnitine	− 0.436030	0.230849	− 1.889	0.0589
	Diglyceride (33:4)	0.194136	0.198864	0.976	0.3290
	Creatine	− 0.001211	0.003222	− 0.376	0.7071
	Tridecanoic acid	− 0.001950	0.003332	− 0.585	0.5584
	Monoacylglycerol (22:6)	0.003391	0.003096	1.095	0.2733
	Dihydroxycholesterol	− 0.327887	0.177616	− 1.846	0.0649
	Biliverdin	− 0.362871	0.179566	− 2.021	0.0433*
Clinical variables and metabolites	Intercept	− 0.330027	0.726670	− 0.454	0.6497
	Sphingosine (d18:2)	− 0.042235	0.271197	− 0.156	0.8762
	Docosenamide	0.004359	0.004724	0.923	0.3561
	Oxo-tricosanoic acid	− 0.002246	0.003257	− 0.689	0.4905
	Tetracosahexaenoic acid	− 0.004089	0.003290	− 1.243	0.2139
	Ketodeoxycholic acid	− 0.321368	0.302763	− 1.061	0.2885
	Stearoylcarnitine	− 0.461926	0.236503	− 1.953	0.0508
	Diglyceride (33:4)	0.184457	0.208359	0.885	0.3760
	Creatine	− 0.001613	0.003291	− 0.490	0.6241
	Tridecanoic acid	− 0.001681	0.003458	− 0.486	0.6268
	Monoacylglycerol (22:6)	0.003439	0.003158	1.089	0.2761
	Dihydroxycholesterol	− 0.307896	0.182783	− 1.684	0.0921
	Biliverdin	− 0.348474	0.181466	− 1.920	0.0548
	Sex	− 0.091159	0.178688	− 0.510	0.6099
	Age baseline	0.142588	0.177915	0.801	0.4229
	HDL Imputed	0.053527	0.188053	0.285	0.7759
	TG Imputed	− 0.223763	0.185640	− 1.205	0.2281
	BMI baseline	0.402986	1.322.298	0.305	0.7605
	Intensity Statin Therapy	− 0.214802	0.174998	− 1.227	0.2197

**Table 3:** Results obtained for the general lineal models (GLM) carried out using the clinical variables alone (GLM1), the metabolites alone (GLM2), the glycated hemoglobin as reference (GLM3), or the combination of the clinical variables and the metabolites (GLM4), and the combination of the clinical variables and the glycated hemoglobin (GLM5)

Model	Sensitivity	Specificity	Accuracy	Threshold
GLM 1	0.5633	0.6454	0.6132	0.4107
GLM 2	0.7887	0.5818	0.6630	0.3642
GLM 3	0.6761	0.7636	0.7293	0.4429
GLM 4	0.8028	0.5545	0.6519	0.3173
GLM 5	0.6197	0.6909	0.6630	0.4223

## Discussion

Our study identified 12 plasma metabolites by O-PLS DA differing between RE and non-RE patients at the baseline of the study; these metabolites were further used to build a score to assess regression probability. This score was significantly associated with a higher probability of T2DM remission. These 12 metabolites, together with the clinical variables previously described, significantly improved the T2DM remission prediction power of the model. However, the prediction capacity of the model with the clinical variables did not significantly improve when the glycated haemoglobin was added. In practice, this could be achieved by analysing a plasma sample from the patients and determining the concentrations of these molecules.

Lifestyle modifications, including the implementation of healthy diets, result in a beneficial effect on T2DM prevention and management [26]. Recent studies indicated that it was possible to induce T2DM remission by weight loss with calorie restriction interventions [27]. Previous studies showed that bariatric surgeries can revert T2DM regulating the glucose in plasma before a significant weight reduction is obtained [9–11].

The importance of the early identification of diabetic patients with a probability of achieving T2DM remission lies in the ability of the β cells to recover long-term functionality after T2DM diagnosis, and before an irreversible stage of β cell dysfunction [9]. Moreover, an effective and efficient therapeutic action focused on disease remission is especially important in individuals with co-occurring acute myocardial infarction and T2DM, who have a higher risk of developing a new cardiovascular event than those without T2DM [28].

In terms of predicting T2DM remission, a variety of scores and variables were previously used to identify subjects with a probability of remission. Classical clinical variables have shown a reduced prediction capacity [29, 30]. Conversely, we have previously reported how the use of miRNAs or gut microbiota proxies improve the predictive power of the clinical variables alone, improving the estimation of the T2DM remission probability induced by the dietary intervention [31–33]. However, to the best of our knowledge, the plasma metabolite profile has not been applied to such end.

This study has shown a metabolic profile associated with T2DM remission in CHD patients. Here, TOF/LS-MS metabolomics at baseline was used to identify which newly diagnosed T2DM patients will benefit from a dietary intervention (a Med or a low-fat diet) to induce the remission of T2DM, with the main difference between the diets in the amount and type of dietary sources of fat and the amount of carbohydrates [20]. Both diets were ethically appropriate for this profile of patients and no further energy restriction or physical activity was implemented.

This profile was characterized by a reduction in 12 metabolites across RE patients identified by O-PLS DA. This model also showed which metabolites found have the strongest influence. These metabolites ranked with a different degree of significance in the model as shown in Additional file 2: Fig. S1. These metabolites, according to the results from the DeLong test, significantly improved the predictive capacity of the clinical variables alone. These metabolites are included in several insulin-related pathways such as sphingolipid metabolism or alpha-linolenic acid and linoleic acid metabolism. Moreover, taken together, these metabolites may be linked to the lipid alterations associated with T2DM. In fact, the derangement of the lipid metabolism is a common complication in T2DM due to the inadequate functioning of key enzymes and pathways as well as the insulin resistance prevalent in these patients [34]. In addition, the association between dyslipidaemia and atherosclerosis is well established, and the composition of lipid particles in diabetic dyslipidaemia has a stronger atherogenic impact on the disease compared to other kinds of dyslipidaemia [35, 36]. Thus, it would be also expected the relationship of these metabolites with lipid alterations considering that the individuals included in this study were CHD patients. Moreover, these disruptions in the metabolite profile in non-RE patients seem to be additional to the dyslipidaemia associated with CHD, which includes hypertriglyceridemia, hypercholesterolemia, and elevated LDL cholesterol [37]. This present study brings insights into the metabolomics modulations occurring during dyslipidaemia.

The O-PLS DA model showed that out of the 12 metabolites identified, tetracosahexaenoic acid (THA), oxo-tricosanoic acid, dihydrocholesterol, and tridecanoic acid were the most discriminant between RE and non-RE; hence, high levels were observed in non-RE patients. In line with this, it is worth mentioning that THA and tridecanoic acid are rare fatty acids. Current literature is contradictory about the beneficial or detrimental effects of rare fatty acids in CHD or T2DM patients [38–40]. In our study, higher levels of THA and tridecanoic acid were observed in non-RE patients. This could be because our population comprises patients with established CHD in which may exist certain alterations in their lipid species profile patients due to the cardiovascular disease.

Dihydroxycholesterol is a metabolite involved in the primary bile acid biosynthesis pathway and derived from cholesterol. This metabolite was identified as an intermediate in C21-Steroid hormone metabolism [41]. Previous studies have shown that plasma concentrations of cholesterol oxidation (ChOx) products (including different forms of hydroxycholesterol) are elevated in DM1 and DM2 patients compared to age-matched subjects without diabetes [42]. In our study, significantly higher levels of dihydroxycholesterol were identified across those individuals who did not return from T2DM with the dietary intervention, which suggests that the lipid metabolism of those individuals is more impaired in non-RE than in RE patients. The relationship between cholesterol oxidized metabolites and lipid metabolism lies in the fact that high levels of glucose seem to promote lipidic accumulation via the DNA CpG methylation in the DNA methyltransferase-1 (DNMT1) promoter region, where cholesterol oxidized metabolites act as mediators [43]. The high levels of glucose increase a nuclear form of oxidized cholesterol (25-hydroxycholesterol) which activates DNMT1 regulating the expression of several genes implicated in the intracellular lipid metabolism in the liver. This process results in the hypermethylation of genes directly implicated in carbohydrate and lipid metabolism of PI3K, cAMP, insulin, insulin secretion, and diabetic and non-alcoholic fatty liver disease signalling pathways. This suggests that the high hydroxycholesterol plasma levels found in our study, in the non-RE group, might be altering the expression of these genes towards a deleterious gene expression profile. This epigenetic regulation of hepatic cell metabolism seems to also have relevance in non-alcoholic, fatty liver disease, and metabolic syndrome [44, 45].

Hepatic lipogenesis can be suppressed by downregulating the gene SREBP1c (trigged by n3 supplementation), implicated in fatty acid biosynthesis. These inhibitory effects in hepatic lipogenesis are associated with a reduction in plasma levels of THA [46]. In our study, we have observed high levels of THA amongst the non-RE group, which suggests a lack of inhibitory feedback in this pathway. This could explain the metabolic resilience to respond to the dietary intervention in these patients, which could be due to the methylation of the promoter region DNMT1. Previous studies from Nagao and colleagues (2003) showed that the depletion in THA was associated with omega 3-PUFAs supplementation. This suggests that the omega 3-PUFAs ingested in the dietary intervention are effective only in patients with low THA levels at baseline. Therefore, the high levels of THA amongst non-RE patients could predispose them towards a higher degree of metabolic impedance, preventing them to respond to the healthy diet administrated which contains 6–8% of total calories as PUFAs. However, this hypothesis needs further evidence.

We also found elevated tridecanoic acid levels in the DM group, a metabolite previously associated with hypoglycaemia, fatty liver, and cardiomyopathies, that are implicated in functions such as oxidation, cell death, and insulin resistance [47]. The latter is especially important as high tridecanoic aid levels were found in the non-RE group, which included the patients who did not reduce their insulin resistance after the ingestion of the healthy diet intervention.

Predicting which patients with CHD can recover from T2DM is crucial since patients with co-occurring CHD and T2DM have a considerably higher risk of developing a new cardiovascular event than those without T2DM. Furthermore, some of the metabolites might be linked to other physiological processes that remain unclear and future research in this field is needed.

In summary, our study provides new plasma biomarkers to predict, in combination with clinical variables, the dietary remission capacity of T2DM patients with co-occurring CHD. Indeed, the addition of the 12 metabolites identified significantly improved the prediction power of the clinical variables alone.

It is also important to mention the limitations of this study. Firstly, this research is based on a long-term, well-controlled dietary intervention, which despite ensuring the quality of the study, may not reflect the level of compliance in a free-living population. The second limitation is that the remission of T2DM was not the primary endpoint of the CORDIOPREV trial, although it was a secondary objective of this study. However, there are no reasons to believe that this randomization would not have worked in such a large subset of participants, taking into account that the baseline characteristics in the groups of patients analysed in the current study according to the diet were similar.

## Conclusions

This study showed the association of a specific metabolic profile in plasma with T2DM remission in patients with CHD in a dietary intervention. These metabolites, combined with clinical variables, could be used to provide in clinical practice more precise therapeutical advice. This new approach will allow the possibility to discern between newly diagnosed T2DM luckily to remit from diabetes following a dietary intervention, from those who will need a more exhaustive treatment such us anti-glycaemic drugs. That would improve the management of these patients and represent a personalized medicine approach. Moreover, our results suggest that lipid metabolism is implicated in the probability to remit from T2DM. The detailed pathway that allows for high glucose (or high dietary glucose) to produce lipids accumulation is not fully understood yet, but early downregulation of liver lipogenesis seems determinant for the metabolism to recover from T2DM. Further investigations should interrogate the potential methylation of the promoter region DNMT1, to unravel whether epigenetic changes may influence the capacity to return from T2DM.

## Supplementary Information

**Additional file 1: Table S1.** Baseline characteristics of the study population.

**Additional file 2: Figures S1, S2. Figure S1.** Ranking with the 12 most significant metabolites derived from the O-PLS DA model. **Figure S2.** Adjusted Cox for the analysis of the patient risk scored grouped in ascending tertiles of glycated haemoglobin.

## Data Availability

The data that support the findings of this study are available from the corresponding author upon reasonable request.

## Abbreviations

ADA: American Diabetes Association
Adipo-IR: Adipose tissue insulin resistance index
AT: Adipose tissue
AUC: Area under the curve
BMI: Body mass index
CHD: Coronary heart disease
CI: Confidence interval
DI: Disposition index
GLM: Generalized linear model
HIRI: Hepatic insulin resistance index
HMDB: Human Metabolome Database
HOMA-IR: Homeostatic model assessment of insulin resistance
HR: Hazard ratio
IR: Insulin resistance
ISI: Insulin sensitivity index
LF: Low-fat diet
MED: Mediterranean diet
MISI: Muscle insulin sensitivity index
non-RE: Patients who remained as diabetics
O-PLS DA: Orthogonal Projection on the Latent Structure Discriminant Analysis
OGTT: Oral glucose tolerance test
PCA: Principal component analysis
RE: Patients who remitted from T2DM
ROC: Receiver operating characteristic
T2DM: Type 2 diabetes mellitus
THA: Tetracosahexaenoic acid
