# Supplementary material for: Metabolomics analysis of type 2 diabetes remission identifies 12 metabolites with predictive capacity: a CORDIOPREV clinical trial study
Source: BMC Med. 2022 Oct 27;20:373. doi: 10.1186/s12916-022-02566-z (PMC9609192; doi:10.1186/s12916-022-02566-z)
Supplement: Supplementary file 1 — Additional file 1: Table S1. Baseline characteristics of the study population. [file 12916_2022_2566_MOESM1_ESM.pdf]

	Responders (n=73)	Non- Responders (n=110)	*p value*
Men/Women	60/13	92/18	0.799
Age (years)	60.8±1.0	59.3±0.9	0.252
Weight (kg)	80.2±1.3	88.4±1.4	<0.001
Body mass index (kg/m^2)	29.9±0.4	32.1±0.4	0.001
Waist circumference (cm)	101±1	108±1	<0.001
Triglycerides (mmol/L)	1.48±0.10	1.69±0.07	0.090
Total-cholesterol (mmol/L)	4.16±0.08	4.31±0.08	0.203
HDL-cholesterol (mmol/L)	1.11±0.03	1.06±0.02	0.141
LDL-cholesterol (mmol/L)	2.31±0.07	2.42±0.07	0.302
C-reactive protein (nmol/L)	37.1±5.3	33.5±3.6	0.558
HbA1c (mmol/mol)	47.8±0.9	50.7±0.9	0.032
HbA1c (%)	6.53±0.08	6.79±0.08	0.032
Glucose (mmol/L)	5.50± 0.09	6.58±0.14	<0.001
Insulin (nmol/L)	64.4± 5.5	93.3±7.8	0.007
HOMA-IR	3.49±0.42	4.84±0.32	0.010
Insulin sensitivity index	3.16±0.20	2.37±0.12	<0.001
Insulinogenic index	0.70±0.19	0.68±0.14	0.921
Hepatic insulin resistance index	1421±168	1970±129	0.009
Muscle Insulin sensitivity index (x10^2)	1.93±0.22	2.20±0.25	0.452
Disposition Index	0.68±0.06	0.43±0.02	<0.001

Table S1. Baseline characteristics of the study population. Data are mean±SEM. Responders group: patients who reverted from type 2 diabetes after 5 years of dietary intervention follow-up. Non-Responders group: patients who remained with type 2 diabetes after 5 years of follow-up. *p*-values were calculated by One-way ANOVA. Gender *p*-value by chi square analysis.
