# Supplementary material for: Metabolomics analysis of type 2 diabetes remission identifies 12 metabolites with predictive capacity: a CORDIOPREV clinical trial study
Source: BMC Med. 2022 Oct 27;20:373. doi: 10.1186/s12916-022-02566-z (PMC9609192; doi:10.1186/s12916-022-02566-z)
Supplement: Supplementary file 2 — Additional file 2: Figures S1, S2. Figure S1. Ranking with the 12 most significant metabolites derived from the O-PLS DA model. Figure S2. Adjusted Cox for the analysis of the patient risk scored grouped in ascending tertiles of glycated haemoglobin. [file 12916_2022_2566_MOESM2_ESM.pdf]

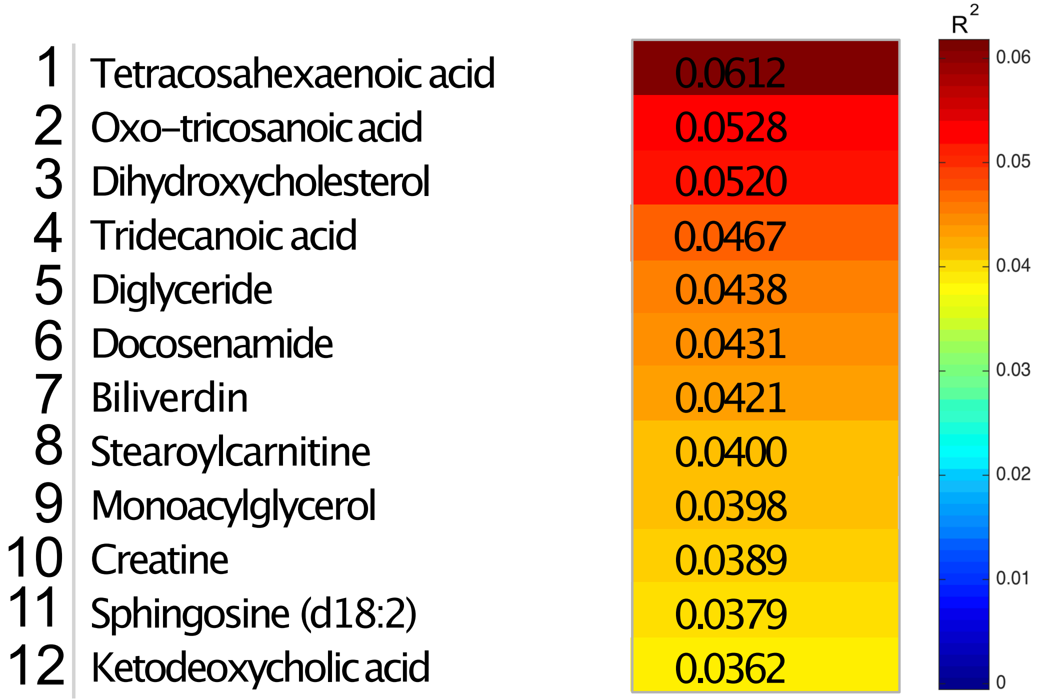

Figure S1. Ranking with the 12 most significant metabolites derived from the O-PLS DA model. They are organised from top to down from the most significant to the least, and the numeric R squared value.

A)

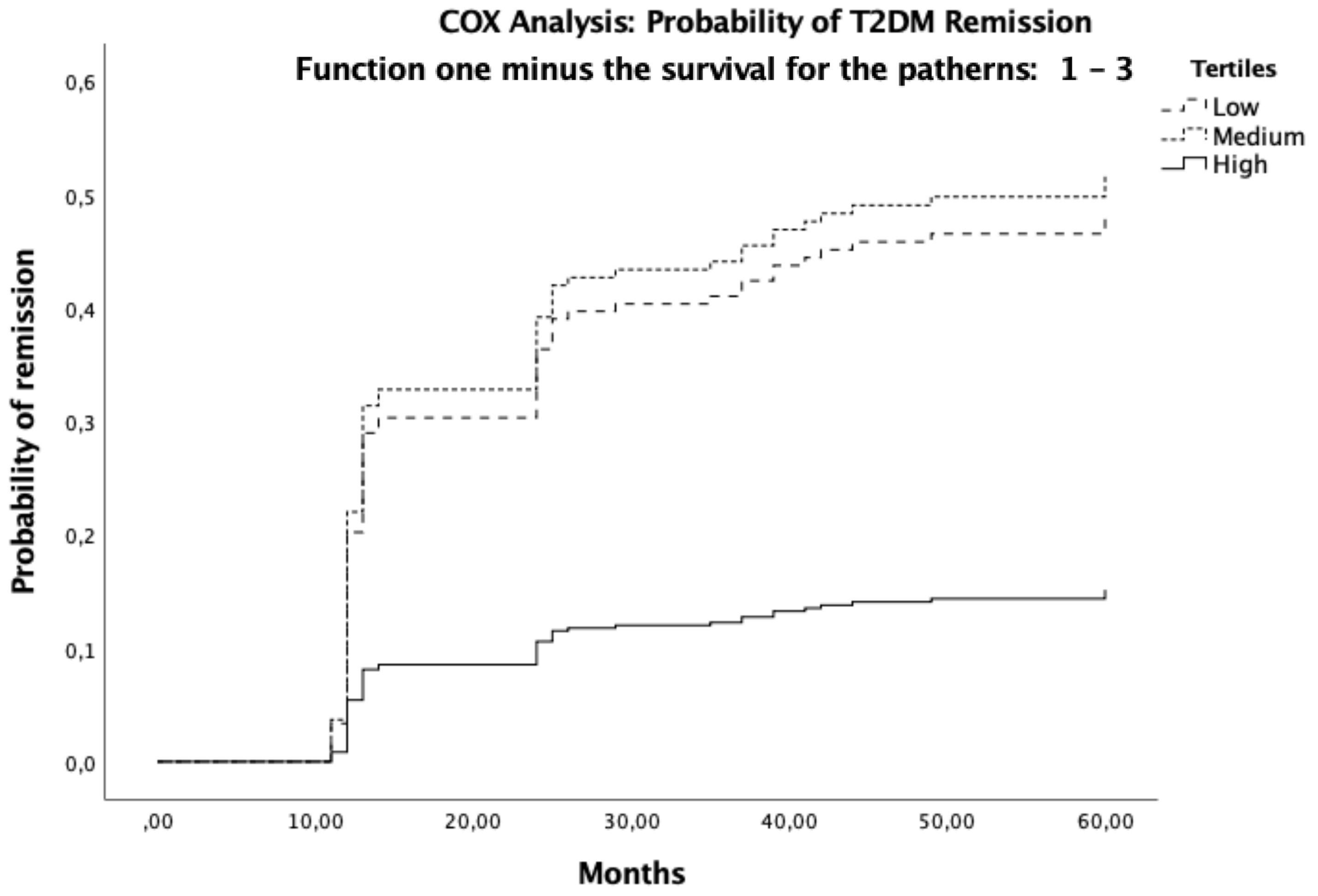

B)

		HR	95.0% CI for HR	
			Lower	Upper
**Tertile 1**	Unadjusted	4.04	1.80	9.05
	Adjusted*	3.80	1.67	8.63
**Tertile 2**	Unadjusted	4.45	1.93	10.25
	Adjusted*	4.31	1.83	10.17
**Tertile 3 (ref.)**		1.00	1.00	1.00

Figure S2. Adjusted Cox for the analysis of the patient risk scored grouped in ascending tertiles of glycated haemoglobin.
